# Supplementary material for: The interactome of intact mitochondria by cross-linking mass spectrometry provides evidence for coexisting respiratory supercomplexes
Source: Mol Cell Proteomics. 2017 Dec 8;17(2):216–32. doi: 10.1074/mcp.RA117.000470 (PMC5795388; doi:10.1074/mcp.RA117.000470)
Supplement: Supplemental Data [file supp_RA117.000470_133922_0_supp_23978_fzffwf.zip › spectra_annotation/mito_spectra_annotation/90-1-8-1-10-1.pdf]

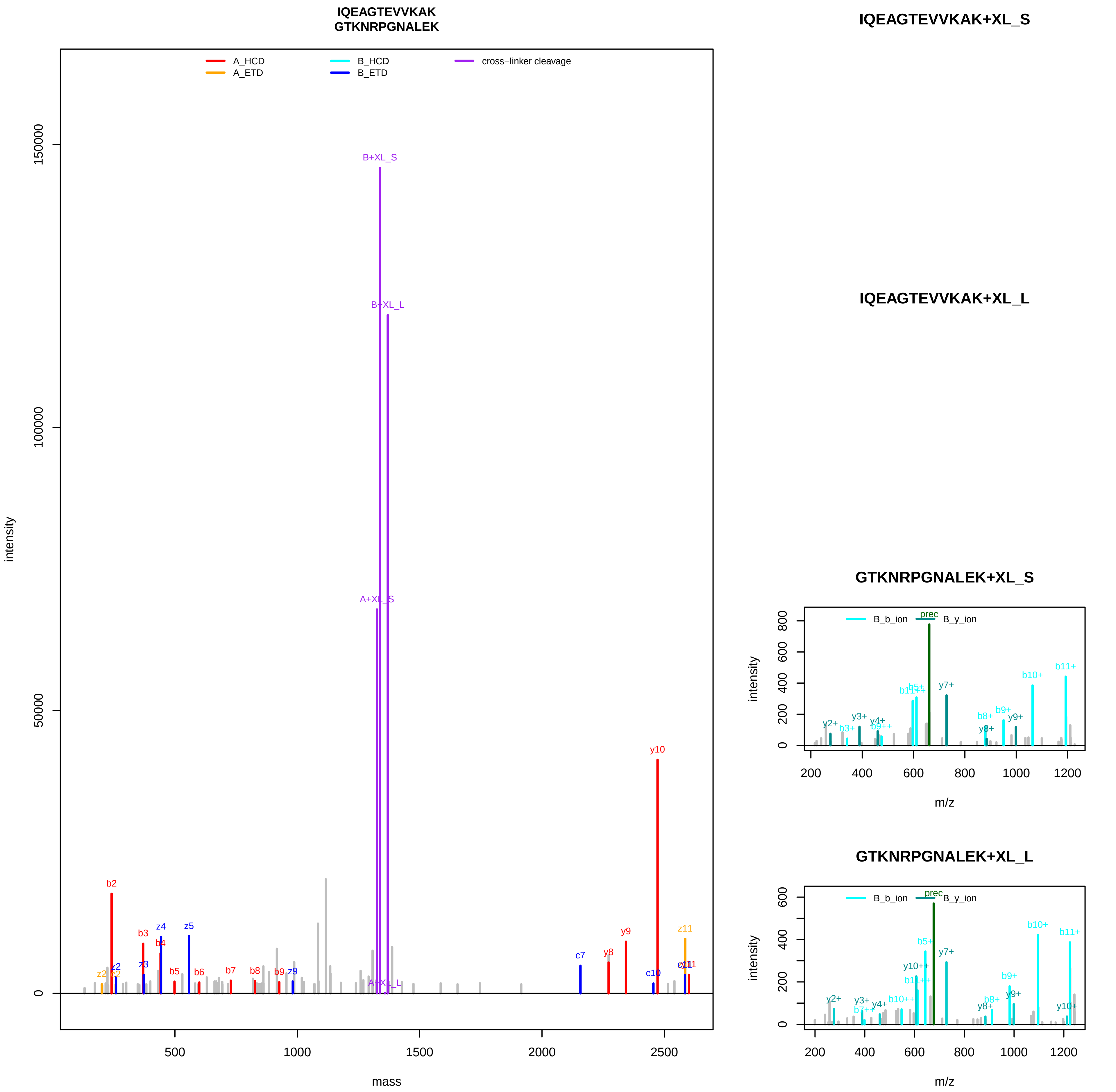

IQEAGTEVVKAK
GTKNRPGNALEK
A_HCD
A_ETD
B_HCD
B_ETD
cross−linker cleavage
B+XL_S
B+XL_L
A+XL_S
A+XL_L
b2
b3
b4
b5
b6
b7
b8
b9
y8
y9
y10
z2
z3
z4
z5
z9
c2
c7
c10
c11
z11
intensity
mass
0
50000
100000
150000
500
1000
1500
2000
2500
IQEAGTEVVKAK+XL_S
IQEAGTEVVKAK+XL_L
GTKNRPGNALEK+XL_S
B_b_ion
B_y_ion
prec
intensity
m/z
GTKNRPGNALEK+XL_L
B_b_ion
B_y_ion
prec
intensity
m/z
